# Depletion of Cellular Iron by Curcumin Leads to Alteration in Histone Acetylation and Degradation of Sml1p in *Saccharomyces cerevisiae*

**DOI:** 10.1371/journal.pone.0059003

**Published:** 2013-03-08

**Authors:** Gajendra Kumar Azad, Vikash Singh, Upendarrao Golla, Raghuvir S. Tomar

**Affiliations:** Laboratory of Chromatin Biology, Department of Biological Sciences, Indian Institute of Science Education and Research, Bhopal, India; Florida State University, United States of America

## Abstract

Curcumin, a naturally occurring polyphenolic compound, is known to possess diverse pharmacological properties. There is a scarcity of literature documenting the exact mechanism by which curcumin modulates its biological effects. In the present study, we have used yeast as a model organism to dissect the mechanism underlying the action of curcumin. We found that the yeast mutants of histone proteins and chromatin modifying enzymes were sensitive to curcumin and further supplementation of iron resulted in reversal of the changes induced by curcumin. Additionally, treatment of curcumin caused the iron starvation induced expression of *FET3, FRE1* genes. We also demonstrated that curcumin induces degradation of Sml1p, a ribonucleotide reductase inhibitor involved in regulating dNTPs production. The degradation of Sml1p was mediated through proteasome and vacuole dependent protein degradation pathways. Furthermore, curcumin exerts biological effect by altering global proteome profile without affecting chromatin architecture. These findings suggest that the medicinal properties of curcumin are largely contributed by its cumulative effect of iron starvation and epigenetic modifications.

## Introduction

The eukaryotic genome is packaged into a highly complex nucleoprotein structure called the chromatin [1], [2]. A precise organization of chromatin is essential for replication, repair, recombination, and chromosomal segregation [3]–[6]. The dynamic changes in the chromatin structure are brought about by post-translational modifications including phosphorylation, acetylation, methylation, ubiquitylation and sumoylation of the amino-terminal tails and globular regions of the highly basic histone proteins by chromatin modifiers [7]–[11]. Among these, acetylation of histone proteins plays a pivotal role in the regulation of gene expression. Abnormal activity of both HATs and HDACs has been associated with the manifestation of several diseases [12], [13]. These enzymes, therefore, are potential new targets for therapy.

Curcumin (1,7-bis (4-hydroxy-3-methoxyphenyl)-1,6-heptadiene-3,5-dione) is the major yellow pigment extracted from turmeric and used extensively in traditional Indian medicine [14]. The chemical structure of curcumin enables it to interact with a large number of molecules inside the cell including transcriptional factors, cytokines, enzymes, growth factors and their receptors leading to a variety of biological effects [15]. Curcumin modulates the cell cycle, growth, induction of differentiation, up-regulation of pro-apoptotic factors, and inhibition of reactive oxygen species production [16]–[18]. Due to these diverse pharmacological effects it is being used to treat cancer, neurodegenerative diseases, arthritis, multiple sclerosis, diabetes type II, cystic fibrosis, asthma etc. [19]–[22]. One possible mechanism by which curcumin might exert its numerous effects is through epigenetic modulation by targeting various epigenetic factors, such as HDAC, HAT, DNMTs, and miRNAs [23]–[27].

The ability of curcumin to induce iron starvation by chelating cellular iron has been well documented [28], [29]. Iron is an essential cofactor for various cellular enzymes involved in most of the major metabolic processes and therefore, is an indispensable micronutrient for all eukaryotic organisms [30]. Iron is a cofactor for ribonucleotide reductase (RNR) complex which catalyzes the *de novo* synthesis of cellular deoxyribonucleotides [31]. The RNR complex is comprised of two major subunits (Rnr1, Rnr3) and two minor subunits (Rnr2, Rnr4) [32], [33]. RNRs regulate the levels of cellular dNTP pools which are required for DNA replication and repair. RNR genes are among the best studied transcriptional targets of DNA replication and damage, regulated by Mec1/Rad53/Dun1 checkpoint kinase pathway [34]. At the post-translational level, the activity of RNR complex is inhibited by Sml1p which binds to Rnr1p. The failure to maintain the dNTP pools and/or their relative amounts results in genetic abnormalities or cell death [35].

Although curcumin is able to directly interact with multiple intracellular signal transduction pathways and other target proteins, accumulating data supports the concept that epigenetic modulation by curcumin might play a major role in transducing its pharmacological property. In spite of the pleiotropic effects reported, mechanism of action of curcumin remains poorly understood. The present study was undertaken to elucidate the molecular mechanism of action of curcumin using yeast as a model organism. To our knowledge, for the first time, we are reporting the action of curcumin is mediated through iron starvation induced epigenetic changes. In addition, our results reveal that curcumin induces degradation of Sml1p. We propose that degradation of Sml1p might be a compensatory mechanism to overcome curcumin induced stress.

## Results

### Increasing dose of curcumin causes reduction of cell viability in yeast cells

**Figure 1:**
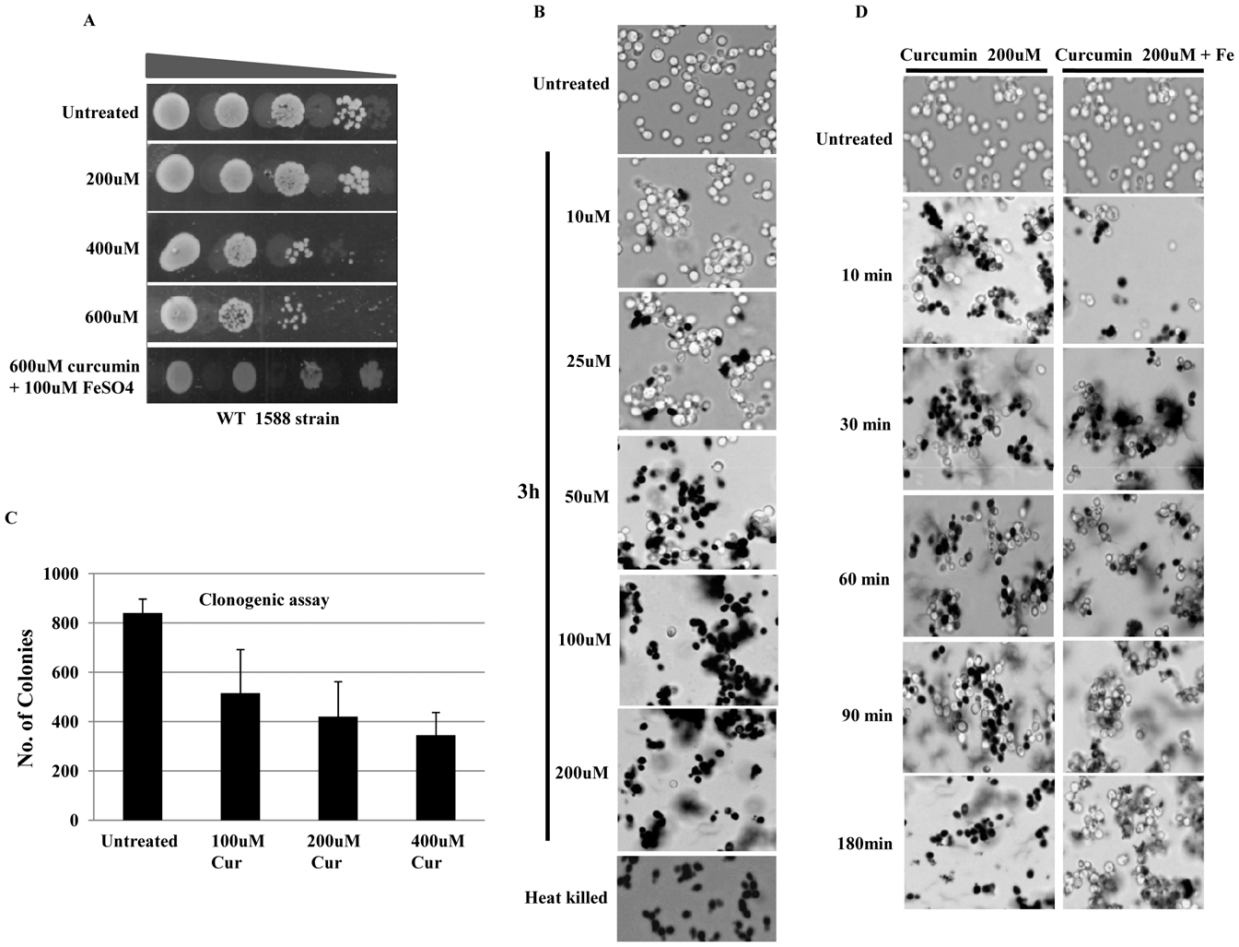
Curcumin inhibits growth of yeast cells and its effect is antagonized by iron supplementation. A) Growth Assay; 1588-4C (Wild-type) was grown up to log phase. 3 µl of each undiluted and 10-fold serially diluted culture was spotted onto control SCA plates, SCA plates containing 200, 400, 600 μM curcumin, and SCA plates impregnated with iron (100 μM) in combination with 600 μM curcumin. All plates were incubated at 30°C for 72 hr and photographed. B) Viability Assay; 1588-4C (Wild-type) was cultured up to mid-log phase and treated with 10, 25, 50, 100 and 200 μM curcumin for 3 hr and cells were stained with 0.3% methylene blue for checking viability. Untreated and heat killed cells were taken as negative and positive controls respectively; viability was observed under light microscope (400X) and photographed. C) Clonogenic assay; equal number of cells from mid-log phase of untreated and methylene blue stained curcumin (400 μM) treated cultures were spread on standard SCA plates in triplicate. All plates were incubated at 30°C and the colony forming ability was analyzed after 36 hr. No. of colonies were counted and shown in the form of bar diagram. D) Viability Assay; 1588-4C (Wild-type) was cultured up to mid-log phase, treated with 200 μM curcumin or iron (100 μM) in combination with 200 μM curcumin. A small number of cells were collected and stained with 0.3% methylene blue at different time points (10, 30, 60, 90 and 120 min) of curcumin treatment for viability examination. Untreated and heat killed cells were taken as negative and positive controls respectively; viability was observed under light microscope (400X) and photographed.

Curcumin is being used as chemotherapeutic agent and it has been well documented as a natural compound having low cellular toxicity. Curcumin is reported to be non-toxic to humans in doses up to 8,000 mg/day when taken orally for 3 months [36]. We have screened the effect of curcumin on yeast cells to determine the dose at which it shows maximum biological effect. To determine the effect of curcumin dosage, we performed growth assay in presence of curcumin at different concentrations (200–600 µM) on SCA (synthetic complete + Agar) media. We found growth inhibition in wild-type yeast cell in dose-dependent manner (Fig. 1A). To identify the effect on cell viability, we carried out viability assays on cells treated with different concentrations of curcumin using the vital dye, methylene blue (Fig. 1B). Results of viability assay suggest that the cells started becoming metabolically inactive or non-viable following curcumin treatment. For further verification, we performed clonogenic assay to discriminate whether stained cells were inactive or inviable. This result was then confirmed by the clonogenic assay. Clonogenic assay is the method to determine cell death or effectiveness of cytotoxic agents [37]. Equal numbers of cells were spread on standard SCA plates in triplicates, our result showed that there was significant decrease in number of colonies compared to curcumin treated samples (Fig. 1c). Previous studies reported that curcumin exerts its biological effects by the chelation of cellular iron [28], [29], [38], [39]. Therefore, we hoped to reverse the effects of curcumin treatment by the addition of iron to the growth medium. We thus conducted viability assays on cells that had been treated with curcumin for 3 hr followed by iron supplementation (for another 3 hr) in the medium. We observed that the effect of curcumin was completely reversed by the addition of iron to culture growth medium (Fig. 1A, D).

### Curcumin mediates its action through histone proteins and chromatin modifying enzymes

**Figure 2:**
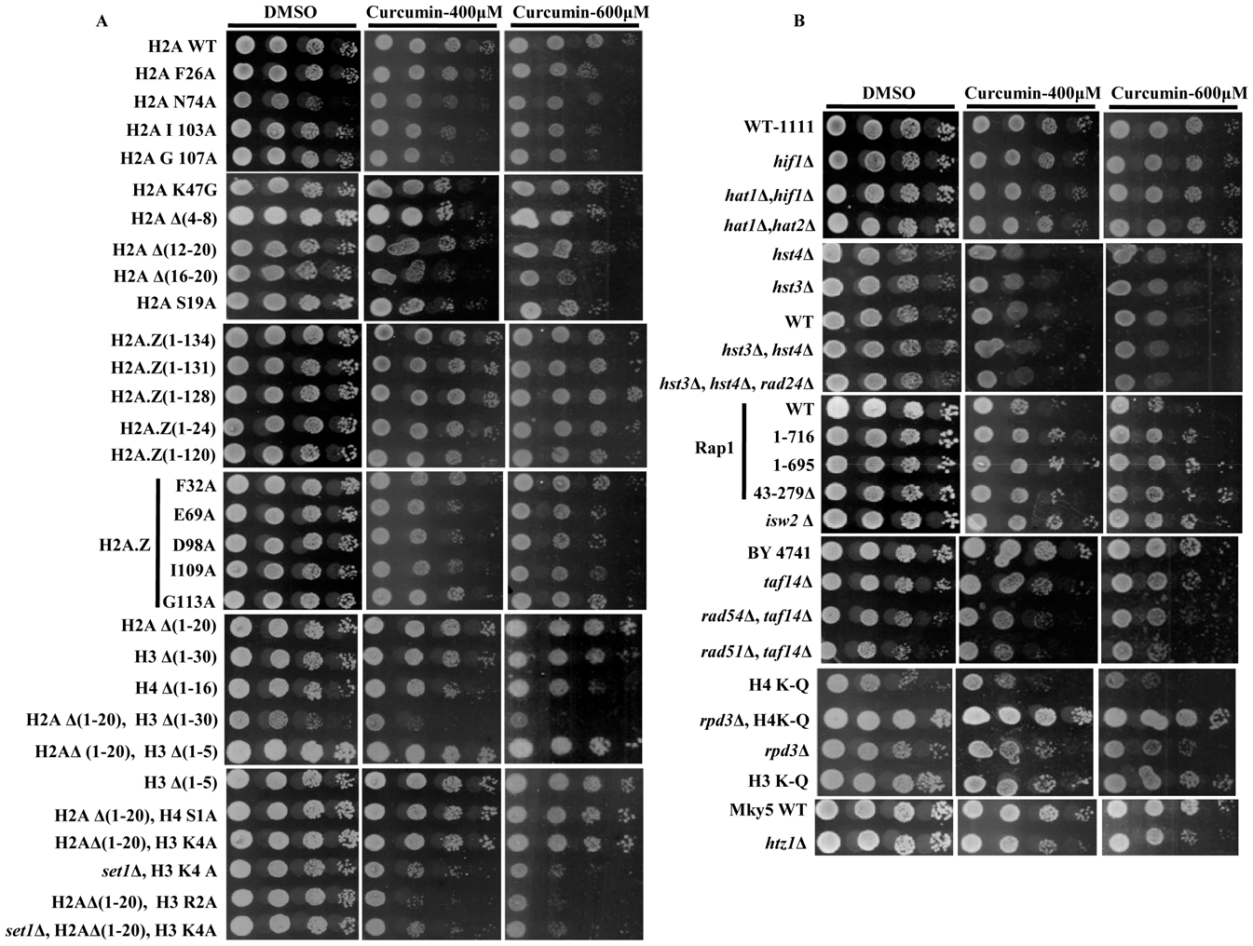
Mutations in histone proteins and chromatin modifiers causes increased sensitivity to curcumin. A & B) Growth Assay; wild-type and different mutant yeast strains were grown up to log phase. 3 µl of each undiluted and 10-fold serially diluted culture was spotted onto control SCA (DMSO) plates or SCA plates containing 400 and 600 μM curcumin. All plates were incubated at 30°C for 72 hr and photographed.

To investigate the effect of curcumin on growth phenotypes of wild-type (WT) and mutant yeast cells, growth assays were performed. We observed that increasing doses of curcumin lead to growth arrest. Availability of various yeast mutants made it possible to identify the molecular mechanism of curcumin action. Histones H3, H2A, H4 and H2A.Z mutants along with some mutants of chromatin modifier proteins were used to examine the effect curcumin on their viability. We found that mutants with histone H2A, H3, or H4 NH_2_-terminal tail truncations showed growth defect in presence of curcumin as compared to wild type (Fig. 2A). N-terminal tails of histone proteins serve as sites for several post translational modifications (PTMs). Removal of these tails mimics a condition in which PTMs cannot take place. The hypersensitivity of these mutants to curcumin suggests that histone N-terminal tails or their respective PTMs plays a critical role in providing resistance to curcumin-induced stress. Similarly, cells with point-mutations in globular region of H2A (F26A, N74A, I103A, G107A) were also found to be sensitive to curcumin, suggesting that the effect of curcumin was mediated and modulated by these residues (Fig. 2A). These mutants were also reported to be sensitive for MMS and caffeine [40]. As canonical H2A mutants showed sensitivity to curcumin, we then also analyzed the sensitivity of histone variant H2A.Z mutants. The canonical H2A histone is substituted by its variant H2A.Z in a non-random manner throughout the genome, but is mainly enriched in promoter regions and is involved in regulation of gene expression [41]. We found that H2A.Z knockout cells were sensitive to curcumin as were some of the strains with point mutations (E69A, D98A and I109A) in the H2A globular region suggesting that curcumin mediates its action through histone H2A.Z variant (Fig. 2A & B) too.

Based on our sensitivity assays, we observed that curcumin functions through histones and histone variant proteins. Histone proteins are post-translationaly modified by various chromatin modifying enzymes which dictate the outcome of gene expression. Therefore, we analyzed mutants of chromatin modifying enzymes for curcumin sensitivity. Particularly, we observed *rpd3* and *taf14* knockout cells to be hypersensitive to curcumin (Fig. 2B). Rpd3p and Taf14p are known to play an important role in DNA damage response [42], [43]. The deletion of *taf14* along with the deletion of *rad54* or *rad51* made strains more sensitive to curcumin. Cells with combinations of H4K-Q (K5, 8, 12, 16Q) point mutations and *rpd3* deletion showed no effect of curcumin on their growth (Fig. 2B) which further suggests that curcumin functions through histone tails. Set1 knockout cells with H2AΔ (1–20), H3K4 point mutations were sensitive to the extent that they exhibited growth arrest. We also screened Rap1, Hat1, Hat2, Hif1, Hst3, Hst4, Rad2, Rad24, and Rad52 protein mutant cells to examine the effect of curcumin but did not observe any significant effect on their growth (Fig. 2B), indicating that curcumin specifically activates signaling pathways independent of these proteins. Taken together, these results demonstrated that curcumin mediates its action through histone proteins and chromatin modifying enzymes.

### Iron supplementation antagonizes the effect of curcumin on yeast cells

**Figure 3:**
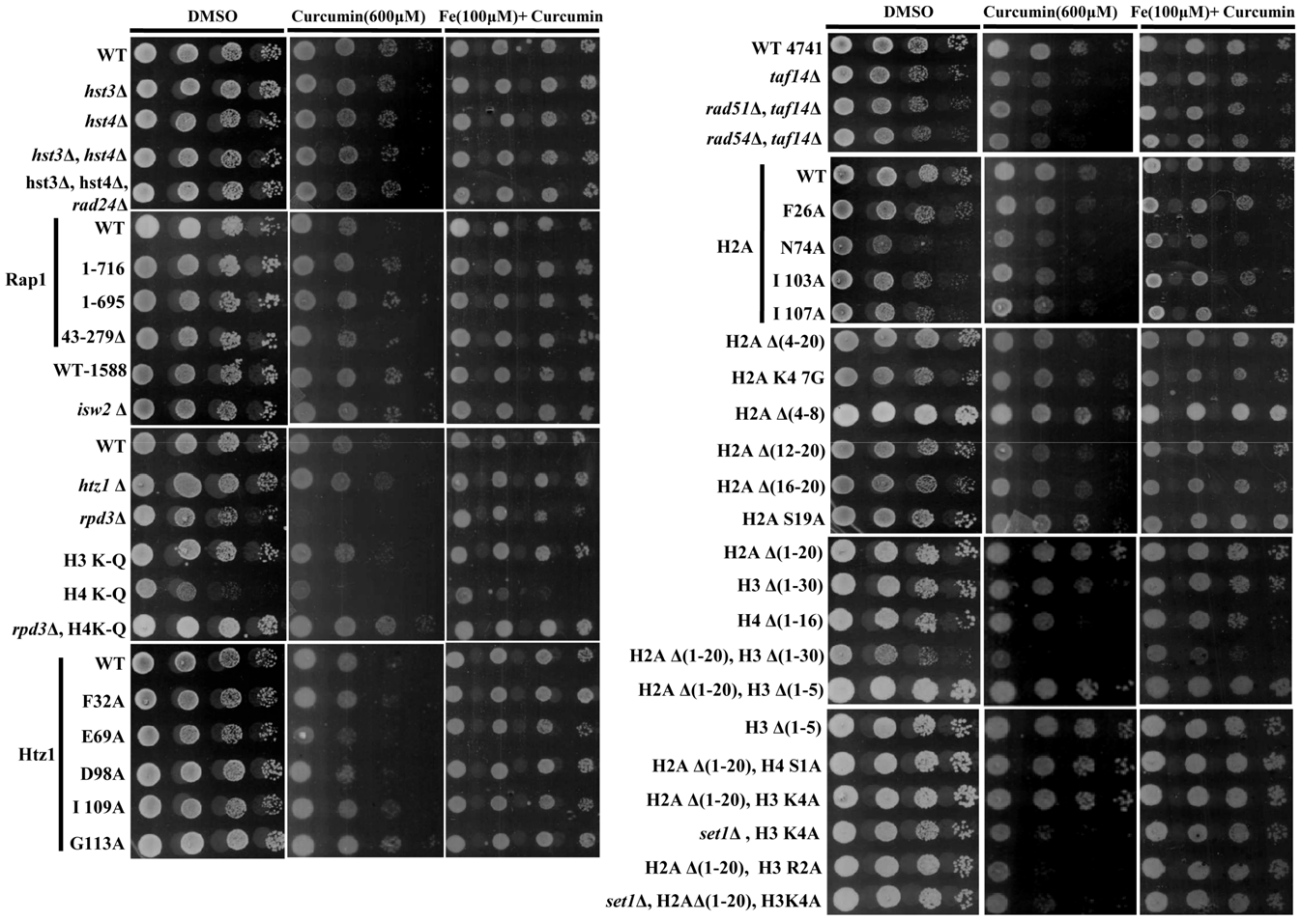
Supplementation of iron suppresses the effects of curcumin. Growth Assay; wild-type and different mutant yeast strains were grown up to log phase. 3 µl of each undiluted and 10-fold serially diluted culture was spotted onto control SCA (DMSO), SCA plates containing 600 μM curcumin and plates containing iron (100 μM) in combination with 600 μM curcumin. All plates were incubated at 30°C for 72 hr and photographed.

It has been reported that curcumin chelates iron leading to reduction in the intracellular level of iron [28]. This property of curcumin may selectively impair specific enzymatic functions by limiting the availability of essential iron cofactors, and altering cellular signaling to produce the diverse physiological effects. Therefore we examined whether the supplementation of iron could rescue the growth defective phenotype due to curcumin. Interestingly, our results showed that iron supplementation diminished the physiological effect of curcumin and normal growth phenotype was recovered (Fig. 3), indicating that curcumin limits cellular iron availability which interferes with the normal functioning of the cells. Importantly, addition of iron to SCA medium at the concentrations employed before (100 µM) had no effect on cell growth in the absence of curcumin (Fig. S1).

### Curcumin inhibits histone H3 K56 acetylation

**Figure 4:**
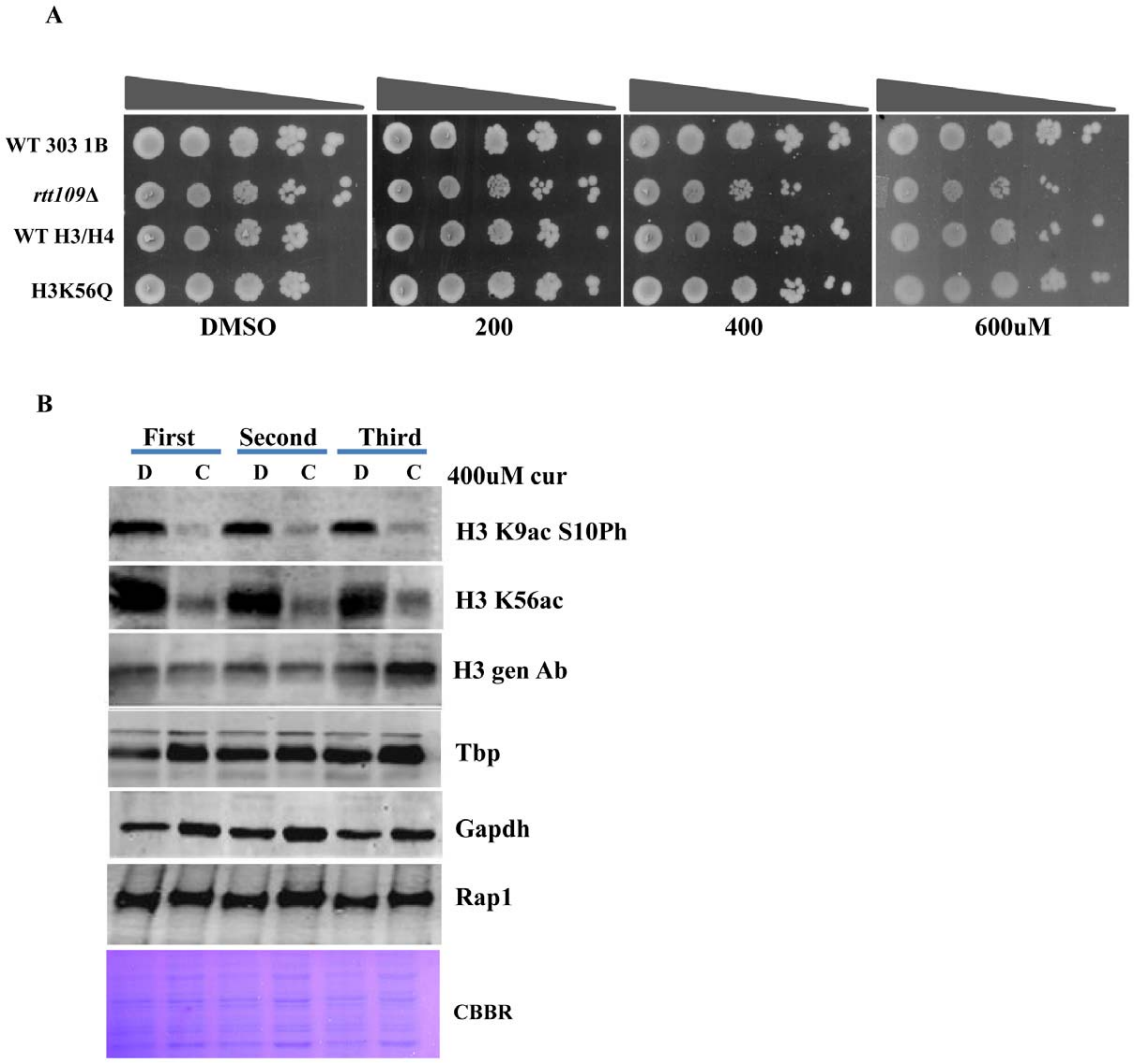
Curcumin reduces H3K56Ac by inhibiting Rtt109. A) Growth Assay; wild-type, Rtt109Δ and H3K56Q mutant yeast strains were grown up to log phase. 3 µl of each undiluted and 10-fold serially diluted culture was spotted onto control SCA (DMSO) plates or SCA plates containing 200, 400 and 600 μM of curcumin. All plates were incubated at 30°C for 72 hr and photographed. B) Wild-type (1588-4C) cells were cultured up to log phase and treated with either DMSO or curcumin (400 μM) for 3 hr in triplicate. Whole cell extracts were prepared by TCA extraction method and samples were subjected to western blot anlaysis using indicated antibodies. Tbp, Gapdh and Rap1 protein levels were used as loading controls.

Curcumin has been reported to act as a HAT and HDAC inhibitor [23] and inhibits the progression of cell cycle by lengthening the G1 phase [28]. Thus, we investigated the sensitivity of various HAT and HDAC knockout (*hat1, hst4, hst3, rtt109, taf14*) yeast strains to curcumin. We found that curcumin selectively inhibits the growth of Rtt109p mutant (Fig. 4A). Rtt109p is a histone acetyltransferase that acetylates H3 at the K56 residue. Further we found the H3K56Q mutant (mimics K56 acetylation) becomes resistant to curcumin suggesting that Rtt109 specifically mediates downstream signaling through acetylating H3 K56. Immunoblotting analysis showed a drastic decrease in the level of H3K56 acetylation and H3K9ac S10 phosphorylation upon curcumin treatment (Fig. 4B). These epigenetic marks are known to be involved in cell cycle progression suggesting that delayed growth phenotypes observed in growth assay might be due to the lack of these epigenetic marks.

### Epigenetic marks altered by curcumin are restored by iron supplementation

**Figure 5:**
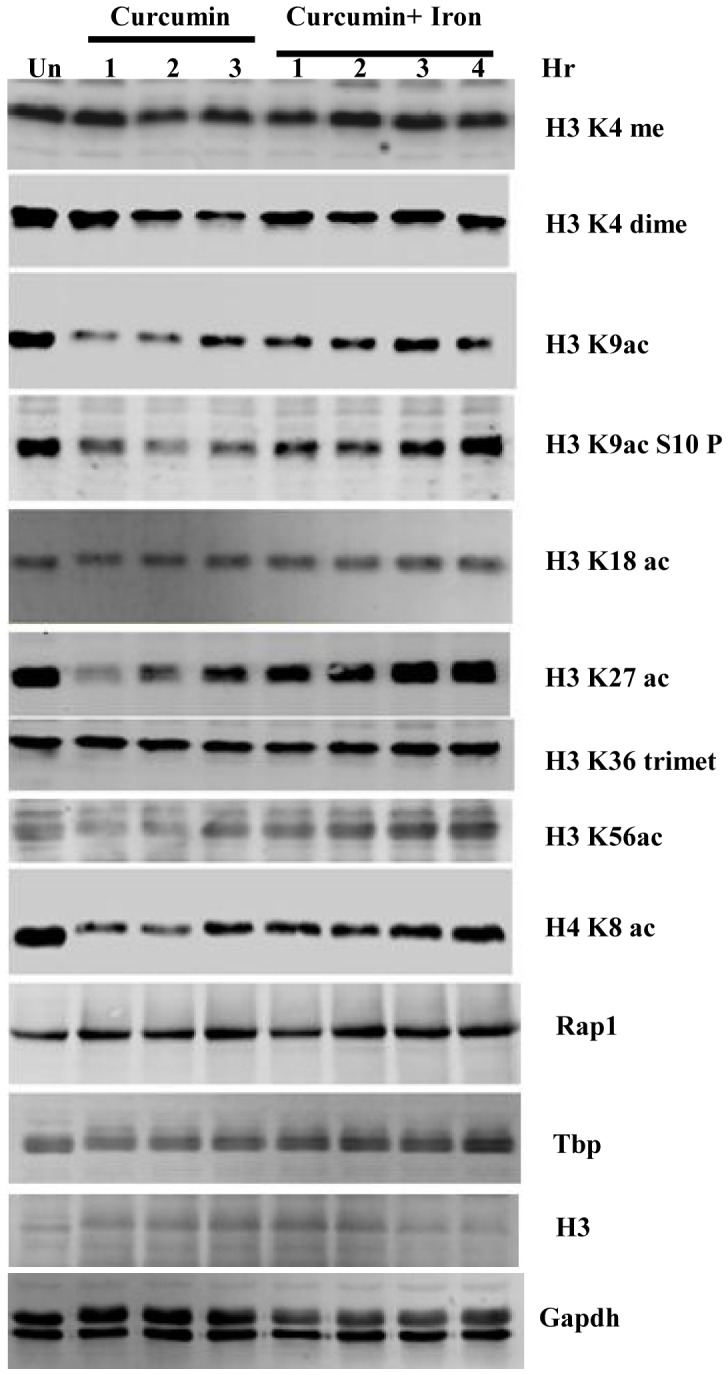
Curcumin induced alterations in epigenetic modifications are restored upon iron supplementation. Wild-type (1588-4C) cells were cultured up to log phase and treated with either DMSO or curcumin (400 μM). Samples were collected after 1, 2 and 3 hr of curcumin treatment. Cultures were supplemented with iron (100 μM) after 3 hr of curcumin treatment. Again samples were collected after 1, 2, 3, 4 hr of iron supplementation. Whole cell extracts were prepared by TCA extraction method and samples were subjected to western blot anlaysis using indicated antibodies. Tbp, Gapdh, H3 and Rap1 levels were used as loading controls. Same extracts were loaded on different gels and transferred separately for western blotting.

Chromatin is a highly dynamic structure. The functional state of chromatin is regulated through post-translational modifications (PTMs) of histones. The balance between histone acetylation and deacetylation is critical for the dynamics of chromatin and gene transcription, deregulation of which has been implicated in various human diseases such as cancer [44]. As curcumin is reported to act as a potent HAT and HDAC inhibitor, we examined global change in histone modifications after curcumin treatment in wild type yeast cells. We found significant decrease in all acetylation marks tested (Fig. S2A). As iron supplementation suppressed the effect of curcumin on cell growth (Fig. 3), we questioned whether epigenetic modifications altered by curcumin treatment can be restored by supplementing iron in the medium of growth. Interestingly, our western blot analysis revealed that curcumin induced epigenetic modifications (H3K9ac, H3K18ac, H3K27ac, H4K8ac and H3K56ac) were indeed restored to normal levels following supplementation of iron (Fig. 5). This observation suggested that curcumin can transiently alter epigenetic marks which can be reversed by supplementing iron.

### Curcumin causes iron starvation which may affect cellular dNTP levels

**Figure 6:**
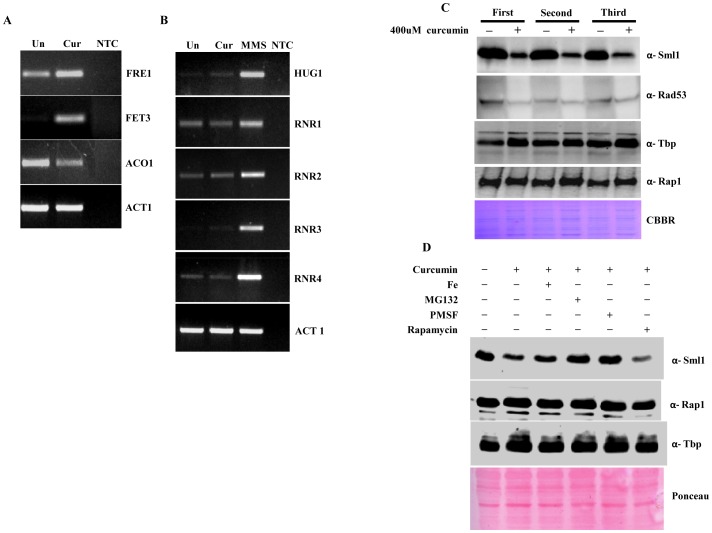
Curcumin induces iron starvation and Sml1p degradation. A & B) RT-PCR Analysis; Logarithmically grown wild-type (1588-4C) cells in standard SC liquid media were treated with either DMSO or curcumin (400 μM) for 3 hr. Total RNA were extracted and reverse transcribed to cDNA. Semi-quantitative PCR analysis was performed to assess the levels of *FRE1, FET3, ACO1, RNR1/2/3/4, HUG1* and *ACT1* transcripts. C) Wild-type (1588-4C) cells were cultured up to log phase and treated with either DMSO or curcumin (400 μM) for 3 hr in triplicate. Whole cell extracts were prepared by TCA extraction method and samples were subjected to western blot anlaysis using antibodies against Sml1, Rad53, Tbp and Rap1. D) Wild-type (1588-4C) cells were grown up to log phase, cultures of cells were divided equally and pre-incubated with either 1 mM PMSF or 100 mM MG132 for 90 min and then treated with curcumin (400 μM) for 3 hr. In addition, some cells were treated with iron (100 μM) and rapamycin (50 ng/ml) in presence of curcumin (400 μM) for 3 hr. Sml1 protein levels were analyzed by western blotting and cellular levels of Rap1, Tbp and Gapdh were used as loading controls.

Based on our observations, we for the first time, found that the sequestering of iron is the key process by which curcumin mediated signaling pathways are activated, and that the effect of curcumin was neutralized in presence of iron. It has been reported that iron is required to maintain normal cellular level of dNTPs [45]. Hence we hypothesized that growth arrest observed in presence of curcumin might be due to decreased dNTP levels which resulted from iron sequestration. It has been shown that curcumin treatment leads to up-regulation of *FET3* and *FRE1* [28]. To confirm our hypothesis, first we conducted the transcriptional analysis of iron regulated genes (*FET3, FRE1* and *ACO1*) after curcumin treatment using RT-PCR. We found significant up-regulation of *FRE1, FET3* and down-regulation of *ACO1* (Fig. 6A), which confirmed that cellular iron starvation followed by curcumin treatment. It is already known that the level of cellular dNTPs depends on the activity of RNR complex which in turn depends on the availability of iron [45]. As our above RT-PCR result revealed that iron starvation occurred following curcumin treatment, we next analyzed the transcriptional levels of RNR genes (*RNR1, RNR2, RNR3,* and *RNR4*). We, however, did not observe any significant increase in RNR transcription upon curcumin treatment (Fig. 6B). Since the activity of RNR complex is also regulated at the post-translational level by Sml1p by inhibiting Rnr1p [46], we examined Sml1p levels after curcumin treatment by western blot analysis and observed a significant decrease (Fig. 6C).

### Curcumin causes degradation of Sml1p through vacuole and proteasome mediated degradation pathway

As we observed significant Sml1p degradation upon curcumin treatment, we suspected that Rad53 phosphorylation, which is responsible for the degradation of Sml1p in response to DNA damage, might be involved too [46]. Our western blot analysis of Rad53 phosphorylation failed to detect any obvious checkpoint activation upon curcumin treatment indicating that the degradation of Sml1p is independent of Rad53 checkpoint kinase pathway or basal activity of Rad53 is sufficient to degrade Sml1 upon curcumin treatment (Fig. 6C). Rad53 phosphorylation is also known to be regulated by the TOR pathway; hence we examined Sml1p levels in the presence of rapamycin after curcumin treatment by western blotting. The result revealed that curcumin induced the degradation of Sml1p in a TOR-independent manner as there was no significant increase in Sml1p level (Fig. 6D). Therefore, we further investigated the involvement of two other obvious pathways of degradation, vacuole and proteasome-mediated degradation pathways. Pre-incubation of yeast cells with inhibitors of proteasomal (MG132) or vacuolar (PMSF) pathways prevented the decrease in level of Sml1p upon curcumin treatment (Fig. 6D) indicating that curcumin induces Sml1p degradation through these pathways.

### Curcumin affects global proteome profile in yeast

**Figure 7:**
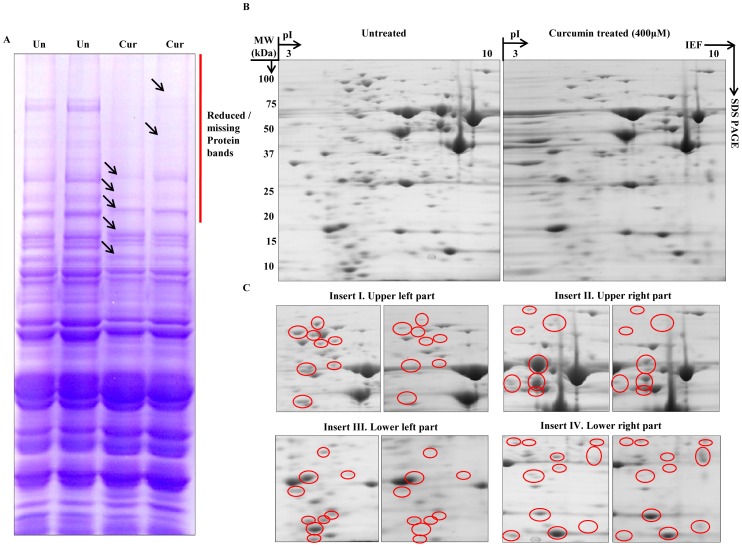
Curcumin alters global proteomics. Whole cell lysate was prepared by disrupting mid-log phase untreated and curcumin (400 μM) treated cultures using chilled glass beads. Protein extracts were prepared by precipitation and clean-up as mentioned in ‘Materials and methods’. A) Equal quantity of protein extracts were resolved by SDS-PAGE. B) 200 µg of total cellular proteins from untreated and curcumin-treated cultures was resolved by two-dimensional gel electrophoresis; first by isoelectric focusing (IEF) on a 7 cm long Immobiline^TM^ pH 3–10 DryStrip, followed by SDS-PAGE. The gel was stained with Coomassie Brilliant Blue R-250. C) Enlarged images of the upper left (Inset I), upper right (Inset II), lower left (Inset III), and lower right (Inset IV) regions of the two-dimensional gels containing proteins from untreated and curcumin-treated cells are shown. The differentially expressed spots are indicated by red circles.

Our analysis of protein extracts on SDS-PAGE revealed significant mobility shift and disappearance of high molecular weight proteins after curcumin treatment (Fig. 7A). Therefore, we were motivated to examine global proteomic changes by performing 2D gel electrophoresis of curcumin treated cell extracts. 2D analyses showed significant changes in protein profile after curcumin treatment (Figs. 7B & C). Proteins showing a correlated difference in expression might be participants in related processes, and as a consequence, might help define the molecular mode of action of curcumin. We are currently in the process of identifying some of the differentially expressed proteins detected after 2D-PAGE through MALDI-MS. All these results put together, one can infer that curcumin probably causes post-translational modifications along with differential expression of certain proteins. The identification of differentially expressed proteins is expected to reveal a fundamental cellular mechanism underlying the action of curcumin.

## Discussion

Curcumin has been the subject of intensive research due to its diverse pharmacological activities. The chemo-preventive and anti-tumor effects of curcumin have been related to its ability to interfere with several signal transduction pathways which are involved in cell proliferation and/or apoptosis [16]. However, these cellular effects are not well established. The present study was carried out to elucidate the fundamental mechanism behind curcumin action using yeast as a model. Our results showed that curcumin causes metabolic inactivation by iron chelation which leads to defective growth phenotype (Fig. 1A & C). The supplementation of iron recovered the normal growth phenotype after curcumin treatment (Fig. 1A & D); hence we propose that the iron molecule plays a major role in transducing downstream signals. To further investigate the mechanism by which curcumin exerted its biological activity, we analyzed the sensitivity of numerous mutants of histone H3, H4, H2A, and its variant H2A.Z to increasing concentrations of curcumin. Interestingly, we found that Histone H3, H4, H2A N-terminal tail truncations lead to significant growth inhibition (Fig. 2A), which suggests that curcumin mediates its action through the N-terminal tail regions of histones. The strains exhibiting combinations of histone tail truncations showed increased sensitivity to curcumin. The effect of point mutations in the globular regions of histone proteins on the sensitivity of mutant yeast cells toward curcumin was also analyzed. Curcumin caused significant growth defect in H2A (F26A, N74A, I103A, G107A) and H2A.Z (E69A, D98A, I109A) point mutants (Fig. 2A). These results suggest that curcumin mediates its action through histone proteins. Histone modifications intersect with cell signaling pathways to control gene expression. Some histone point or partial deletion mutants exhibited increased sensitivity to curcumin. This sensitivity could be due to the effect of curcumin on histone modification as shown in figure 5. Histone tails and its modifications regulate diverse biological processes including transcription, DNA repair, recombination, cell division, differentiation etc [11], [47], [48]. It is also possible that curcumin may disrupt some cellular processes that functions parallel with histone modification. One possible mechanism by which curcumin might exert its numerous effects is through epigenetic modulation by targeting various epigenetic factors, such as HDAC, HAT, DNMTs, and miRNAs [23]–[27] which regulates various cellular pathways.

Our results from growth assay of histone tail mutants motivated us to investigate the effect of curcumin in absence of chromatin modifier proteins (*anc1, rad51, rad54, rpd3, set1, rtt109*). We found that *rpd3*Δ cells were significantly sensitive to curcumin (Fig. 2B), a result which correlates with the previously described observation that Rpd3p is required for growth under iron limiting condition [49]. The H4K-Q mutant also showed sensitivity for curcumin while we observed H4K-Q *rpd3*Ä double mutant cells showed better growth than each single mutant (Fig. 2B). This result correlates with earlier reported observation [50] that for many genes which are repressed by Rpd3p, acetylation of either the H3 or the H4 amino terminus could suffice to overcome this repression. Probably in our case similar phenomenon took place; curcumin effect is antagonized when *rpd3*Ä and H4 (K5, 8, 12,16Q) are present together compared to each single mutant as seen in spot test assay (Fig. 2B). As we have observed that some of the histone and chromatin modifiers mutant exhibited increased sensitivity towards curcumin, the reason might be that those mutants have altered histone protein levels upon curcumin treatment. Hence we analyzed levels of histone H3 by western blotting and failed to detect any change (Figure S3). Yeast cell knockout of *rtt109*, which acts as a histone acetylase for H3K56, exhibited significant sensitivity to curcumin (Fig. 4A). Moreover, our western blot analysis showed a reduction in H3K56 acetylation (Fig. 4B). This result was confirmed by the growth-resistant phenotype exhibited by H3K56Q mutant in the presence of curcumin (Fig. 4A). Taken together, these results indicate that the acetylation of H3 K56 by Rtt109p drives the yeast cells to survive in presence of curcumin.

The majority of epigenetic Post translational modifications (PTMs) including acetylation, methylation, phosphorylation etc. are known to be localized on the tails of histone proteins [51], [52]. Given the significance of histones and their respective tail domains in epigenetic modulations, it became essential for us to decipher the role of histone tails in delineating the mechanism by which curcumin exerts its biological effect. Our western blot analyses revealed that curcumin reduces acetylation marks to a greater extent as compared to methylation marks indicating that it acts as a pan-HAT inhibitor *in vivo* in yeast (Fig. S2A). Chromatin architecture is defined, to a large extent by different PTMs, chromatin modifiers and the incorporation of histone variants. Since curcumin is known to cause histone hypo-acetylation which accounts for the loss of cell viability [53], [54], these results open a new door for us to understand the molecular mechanism involved in the *in vivo* function of curcumin. The accumulated literature on histone acetylation reveals that the extent of acetylation defines various transcription states [55]. Expectedly, the curcumin-induced drastic decrease in acetylation marks leads to an altered protein profile validated by our 2D analysis (Fig. S2A & Fig. 7b).

Chromatin modifying enzymes regulate chromatin structure and function by changing its accessibility to *cis-* and *trans-* acting factors, especially PTMs, which are important events in the regulation of gene expression [56]. The chromatin modifying enzymes such as HDAC, HAT, HMTases, and DNMTs are targets of various drugs that mediate their action by inhibiting these enzymes. It has been well documented that curcumin antagonizes yeast growth by chelating iron [28]. In our present study, supplementation of iron rescued the cells from the growth arrest phenotype. Surprisingly, for the first time, we found a novel way to reset epigenetic marks back to their normal levels by iron supplementation in the presence of curcumin (Fig. 5). Cumulatively, these findings suggest that the alteration in the epigenetic status of host cells by curcumin is reversible. This reversibility can be harnessed during the usage of curcumin in the treatment of various diseases.

Iron is an essential cofactor for enzymes involved in most of major metabolic processes in the cells. It is also known to be crucial for the activity of the Rnr complex [31], [49]. This led us to the hypothesis that the growth sensitivity observed after curcumin treatment might be due to decreased RNR activity, and as a result, reduced dNTP levels. Our results from RT-PCR analysis confirmed our hypothesis to the extent that we know for sure that curcumin causes iron starvation which is reflected in the transcriptional alteration of iron regulated genes like *FET3, FRE1* and *ACO1* (Fig. 6A). On the other hand, our transcriptional analysis of RNR genes revealed that iron starvation did not induce *RNRs* expression (Fig. 6B). Surprisingly, our western blot analysis showed significant degradation of Sml1p in yeast cells following curcumin treatment (Fig. 6C). Under normal growth conditions Sml1p remains associated with Rnr1 to inhibit the formation of the active RNR complex which is necessary for synthesis of dNTPs [51]. The cells lacking sml1p are reported to have higher levels of dNTPs, independent of the increase in RNR transcription [57]–[59]. Similarly, over-expression of RNR1 leads to up-regulation of Sml1p [60], suggesting that Rnr1p and Sml1p level are directly proportional to each other. After curcumin treatment Sml1p level is drastically reduced, we propose that loss of Rnr1 inhibition after Sml1 degradation allows the formation of an active RNR enzyme. Sml1p is known to get sequentially phosphorylated by Dun1 and degraded by a proteasome-mediated pathway during genotoxic stress [46]. Results from present study divulge that curcumin induces sml1p degradation through both the proteasome and the vacuolar degradation pathways (Fig. 6d).

**Figure 8:**
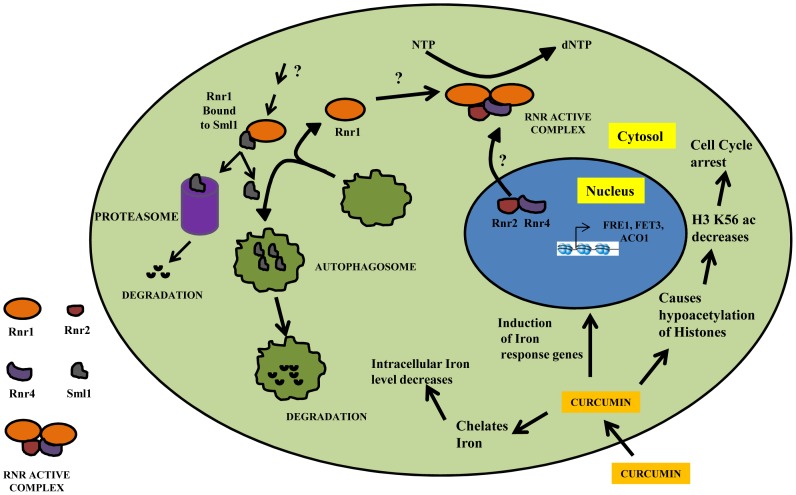
Proposed model for action of curcumin in budding yeast . Curcumin causes iron starvation by chelating it which leads to transcriptional alteration of iron-regulated genes. Histone hypo-acetylation by curcumin results in delayed growth phenotype. Curcumin induces Sml1p degradation through vacuole and proteasome-mediated protein degradation pathways. We propose that after Sml1p degradation the released Rnr1 associates with Rnr2 and Rnr4 to form an active RNR enzyme allowing the production of dNTPs.

Taken together, our results can be summarized in a model (Fig. 8), which describes the molecular mechanism of action of curcumin in budding yeast. Histone proteins and chromatin modifiers are major targets of curcumin activity. After entering the yeast cells, curcumin causes iron starvation. We propose that this phenomenon may result in decreased dNTP levels due to iron limitation which inhibits the RNR complex. Our above hypothesis is partly supported by curcumin-induced Sml1p degradation. Altogether, loss of Rnr1 inhibition after Sml1p degradation allows the formation of an active RNR complex which may further compete for the available iron in order to increase the production of dNTPs so that normal cell cycle progression can be facilitated. Upstream signaling and the physiological significance of curcumin-induced Sml1p degradation is yet to be established. Although this study suggests that curcumin induces iron starvation, its effect on dNTPs levels remains to be answered.

## Materials and Methods

### Strains, chemicals, growth media and conditions

The *Saccharomyces cerevisiae* strains used in this study are listed in Table S1. Media components, chemicals, antibodies and all other reagents used in this study were of molecular biology grade and purchased from Sigma-Aldrich, Merck, HiMedia, GE Healthcare, Invitrogen, Thermo Scientific, New England Biolabs and R&D systems. Unless stated otherwise, yeast cells were grown at 30°C in standard Synthetic Complete (SC) liquid media. For solid Synthetic Complete Agar (SCA) media, 2% Bacto-agar was used in addition to components of SC liquid media.

### Growth assay and viability analysis

To investigate the biological effect of curcumin on the growth of yeast cells, growth assay was carried out by spot tests (as described earlier [61]) using serial dilutions of mid-log phase cultures of wild-type and different mutant strains listed in table S1. Three microliter of each undiluted and 10-fold serially diluted cultures were spotted onto solid SCA plates containing vehicle DMSO or different concentrations of curcumin (400 and 600 ìM). Additionally, some of sensitive strains were spotted on to SCA plates impregnated with both curcumin (600 ìM) and iron (100 ìM). The concentration of iron (100 ìM) used was not toxic to cells (supplementary material). All plates were incubated at 30°C and growth of the yeast strains was recorded at periodic time intervals of 24, 48 and 72 hr by scanning the plates using a HP scanner. In addition to effect of curcumin on growth, the viability of yeast cells was analyzed by 3.7% buffered methylene blue (vital dye) staining after 3 hr of treatment with different concentrations of curcumin [62]. Cells stained dark blue were considered metabolically inactive or nonviable. Dark blue stained heat killed cells were used as a positive control. The viability of yeast cells was recorded by using a LEICA DM500 microscope (installed with the LAS EZ-V1.7.0 software) at 400X total magnification.

### Clonogenic cell survival assay

This clonogenic assay was carried out to discriminate between yeast cells which took up the methylene blue stain (dead cells) and which did not (metabolically active) after curcumin treatment. Equal number of mid-log phase cells from untreated (DMSO) and curcumin (400 ìM) treated cultures were spreaded on standard SCA plates in triplicate after 3 hr of treatment. The plates were incubated at 30°C and survivability was analyzed after 36 h of incubation.

### Western blot analysis

Yeast mid-log phase cultures were treated with vehicle (DMSO) or curcumin (400 ìM) for 3 hr and harvested. The cell pellets were washed with chilled water and subsequently stored at −80°C until protein extracts were made. Whole cell protein extracts were obtained from the frozen yeast cell pellets by 20% trichloro acetic acid (TCA) precipitation following standard protocol [63], [64]. The protein extracts were mixed with equal volume of 2X SDS-PAGE sample loading dye, boiled for 5 min, debris pelleted and the supernatant resolved by electrophoresis on a 10% SDS-polyacrylamide gel. For Immunoblotting analysis, whole cell protein extracts were transferred to Polyvinylidene difluoride (PVDF) membranes using a Bio-Rad mini wet transfer apparatus (Bio-Rad, Hercules, CA, USA). The membranes were then blocked with Odyssey blocking buffer (LI-COR® Biosciences) for 45 min followed by incubation in primary and secondary antibodies for 1 hr each. IRDye® 800CW anti-Rabbit IgG (diluted 1∶15,000; LI-COR® Biosciences) was used as secondary antibody. Blots were scanned by using Odyssey Infrared imager (LI-COR^®^ Biosciences). Following primary antibodies were used: General H3 (Sigma, H0164), H3K4me1 (Abcam, 8895), H3K4me2 (Abcam, 32356), H3K9Ac (Abcam, 69830), H3K9acS10P (Sigma, H9161), H3K18ac (Cell signaling, 9675), H3K27ac (Abcam, 45173), H3K36me3 (Sigma, SAB4800028), H3K56Ac (Sigma, SAB4200328), H4K8ac (Abcam, 15823), Rnr1 (Agrisera, ASO9 576), Rnr2 (Agrisera, ASO9 575), Sml1 (Agrisera, AS10847), GAPDH (Abcam, 37168), and Rad53 (Santa Cruz Biotechnology Inc., SC-6749). Polyclonal antibodies against recombinant TBP and RAP1 were raised in rabbit. Rad53 signals were detected by using HRP conjugated secondary antibodies and ECL reagents (GE Healthcare, place) and chemiluminiscence was captured by Fuji gel-dock system (LAS-4000 mini).

### Reverse Transcriptase-PCR

Total cellular RNA was extracted from mid-log phase wild-type yeast cultures which were left untreated or treated with curcumin (400 μM) for 3 hr as described [65]. The cDNA was synthesized using High Capacity RNA-to-cDNA™ Kit (Life Technologies Corporation, California) according to the manufacturer's instructions. 2 μg of total RNA was used for synthesizing cDNA. The cDNA was amplified by PCR using Taq DNA Polymerase (New England Biolabs), and reverse and forward primers complementary to coding regions of *RNR1, RNR2, RNR3, RNR4, HUG1, FET3, FRE1, ACO1* and *ACT1* (table S2). The PCR amplified products were electrophoresed, stained with ethidium bromide, visualized and photographed.

### Protein degradation analysis

The degradation of Sml1p after curcumin (400 μM) treatment was analyzed in the presence of proteasome inhibitor MG132, the vacuolar inhibitor PMSF, Iron (FeSO_4_) or Target of Rapamycin (TOR) inhibitor each separately. Wild-type 1588-4C cultures were grown at 30°C to 5×10^6^ cells/ml in standard SC media, were split into equal volumes and pre-incubated with either 100 μM MG132, 1 mM PMSF or vehicle DMSO (control) for 90 min. prior to treatment with curcumin (400 μM) as described earlier [66]. The cells were harvested after a 3 hr treatment with curcumin; whole cell protein extracts were obtained as described above using 20% trichloroaceticacid (TCA) and subjected to Western blot analysis.

### Two-dimensional gel electrophoresis

Whole cell lysate was prepared by disrupting cells from mid-log phase untreated and curcumin (400 μM) treated cultures in lysis buffer (50 mM Tris-HCl pH 7.5, 0.6 M NaCl, 0.1% Triton X-100, 5% (w/v) glycerol, 5 mM EDTA, 5 mM DTT, 0.5 mM PMSF and protease inhibitor cocktail) using chilled 400–600 µl of glass beads (Sigma, St. Louis, U.S.A.). To the lysates 0.1 volumes of the solution containing 1 mg/ml DNase I, 0.25 mg/ml RNase A and 50 mM MgCl_2_ was added followed by incubation on ice to remove nucleic acid contamination. Protein extracts were prepared by precipitation using 2-D Clean-Up kit (GE Healthcare) according to manufacturer's instructions. 2D-Gel Electrophoresis was performed as previously described with certain modifications [67]. The first-dimensional isoelectric focusing (IEF) was carried out on rehydrated commercial 7 cm long Immobiline^TM^ pH 3–10 DryStrip (GE Healthcare) with a maximum current limitation of 50 µA/strip in a Ettan-IPGphor® Isoelectric focusing unit (GE Healthcare). After focusing, immobilized pH gradient strips were reduced (2% dithiothreitol) and then alkylated (2.5% iodoacetamide) for 15 min in equilibration solution (6.0 M urea, 75 mM Tris-Cl, pH 8.8, 30% (w/v) glycerol, 2% (w/v) sodium dodecyl sulfate (SDS), and 0.002% Bromophenol Blue) [68]. Gels were subjected to second-dimensional SDS-polyacrylamide gel electrophoresis (SDS-PAGE) on 12% linear polyacrylamide gels. The gels were stained with Coomassie Brilliant Blue R-250 (Sigma) for 3 hr, and then destained. Images were processed using ImageMaster™ 2D Platinum software. Differentially expressed protein spots were analyzed.

## Supporting Information

Figure S1 **Supplementation of iron in absence of curcumin had no effect on cell growth.** Growth Assay; Wild-type and different mutant yeast strains were grown up to log-phase. 3 µl of each undiluted and 10-fold serially diluted culture was spotted on to control SCA (DMSO) or SCA plates containing iron (100 μM).
(TIF)

Figure S2 **Curcumin causes alteration of global epigenetics.** A) Wild-type (1588-4C) cells were cultured up to log-phase and treated with either DMSO or Curcumin (400 μM) for 3 hr in triplicate. Whole cell extracts were prepared by TCA extraction method and samples were subjected to Western blot anlaysis using indicated antibodies. B) Wild-type (1588-4C) cells were cultured up to log phase and treated with either DMSO or curcumin (400 μM). Samples were collected after 1, 2 and 3 hr of curcumin treatment. Cultures were supplemented with iron (100 μM) after 3 hr of curcumin treatment. Again samples were collected after 1, 2, 3, 4 hr of iron supplementation. Whole cell extracts were prepared by TCA extraction method and samples were subjected to Western blot analysis using indicated antibodies.
(TIF)

Figure S3 **Histone H3 level remains same in various mutants upon curcumin treatment.** Wild-type and various mutants shown in figure were cultured up to log-phase and treated with either DMSO or Curcumin (400 μM) for 3 hr. Whole cell extracts were prepared by TCA extraction method and samples were subjected to Western blot analysis using indicated antibodies.- and + indicates untreated and curcumin treated samples respectively.
(TIF)

Table S1 **List of yeast Strains used in this study.**
(DOC)

Table S2 **List of oligonucleotides used in this study.**
(DOC)
