# Correction: Depletion of Cellular Iron by Curcumin Leads to Alteration in Histone Acetylation and Degradation of Sml1p in *Saccharomyces cerevisiae*

**DOI:** 10.1371/annotation/ac974676-18a6-4aee-b38e-57a1142a3dc1

**Published:** 2013-10-25

**Authors:** Gajendra Kumar Azad, Vikash Singh, Upendarrao Golla, Raghuvir S. Tomar

The sixth sentence in the legend for Figure 1 is incorrect. The complete, correct Figure 1 legend is: "C) Clonogenic assay; equal number of cells from mid-log phase of untreated and methylene blue stained curcumin (100-400 μM) treated cultures were spread on standard SCA plates in triplicate."

*rpd3A* appears incorrectly in the Discussion section. The correct name is *rpd3Δ*.

The proper unit of concentration appears incorrectly as ìM in the Material and Methods section. The correct unit is μM.

In the Author Contributions section, Upendarrao Golla (UG) should be listed as one of the authors who performed the experiments. 

