# Supplementary figures and images for: Depletion of Cellular Iron by Curcumin Leads to Alteration in Histone Acetylation and Degradation of Sml1p in Saccharomyces cerevisiae
Source: PLoS One. 2013 Mar 8;8(3):e59003. doi: 10.1371/journal.pone.0059003 (PMC3592818; doi:10.1371/journal.pone.0059003)

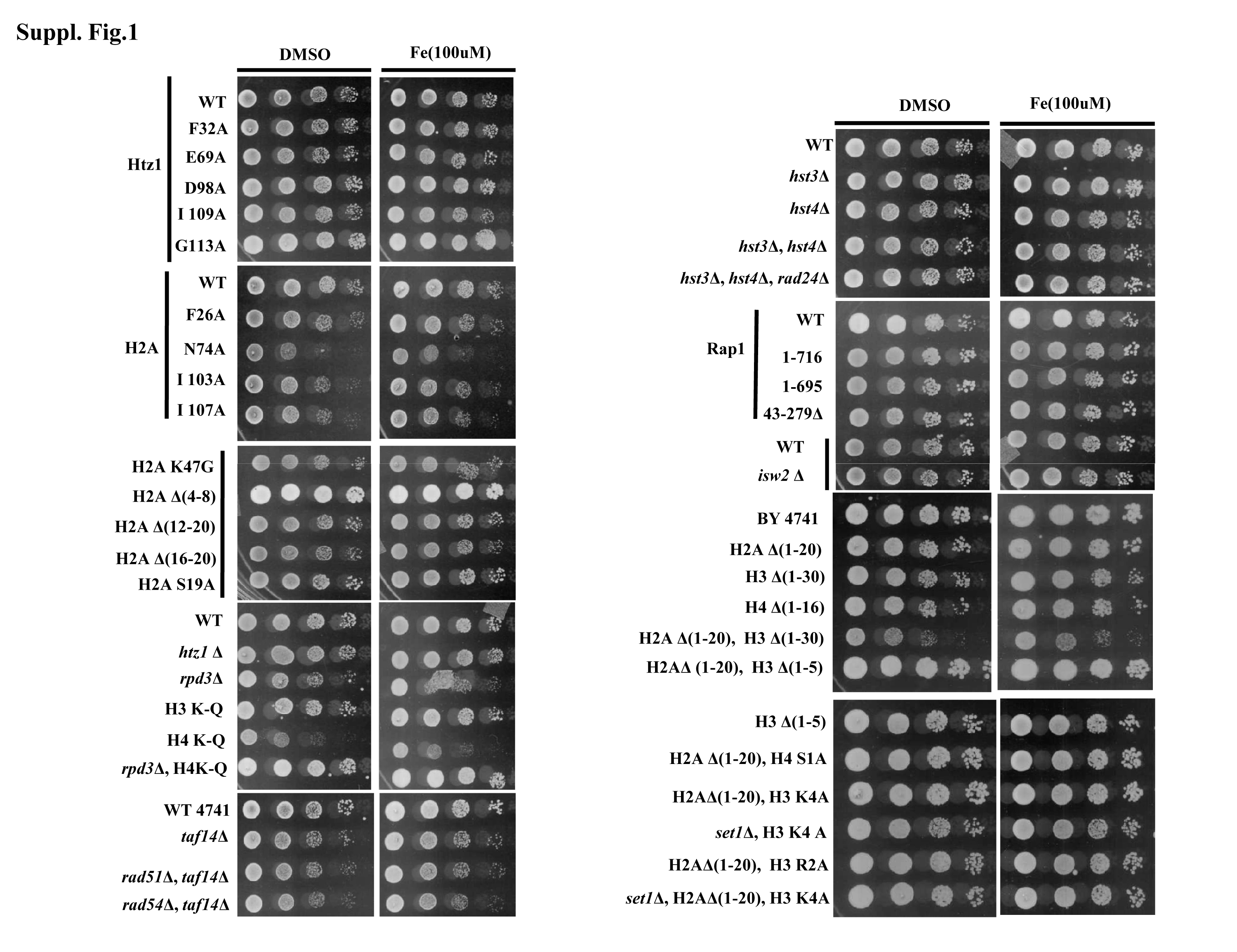

Supplement: Figure S1 — Supplementation of iron in absence of curcumin had no effect on cell growth. Growth Assay; Wild-type and different mutant yeast strains were grown up to log-phase. 3 µl of each undiluted and 10-fold serially diluted culture was spotted on to control SCA (DMSO) or SCA plates containing iron (100 μM). (TIF) [file pone.0059003.s001.tif]

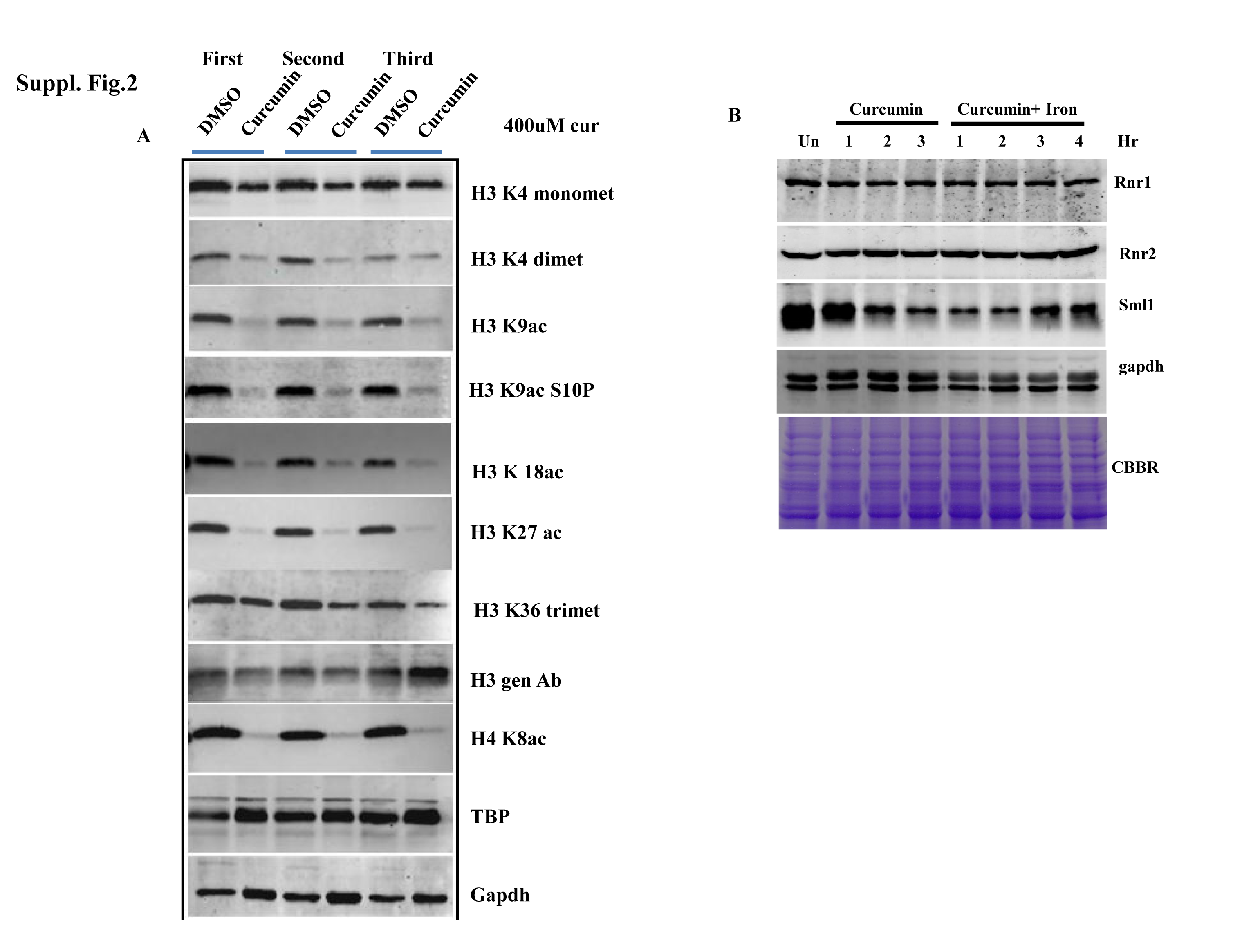

Supplement: Figure S2 — Curcumin causes alteration of global epigenetics. A) Wild-type (1588-4C) cells were cultured up to log-phase and treated with either DMSO or Curcumin (400 μM) for 3 hr in triplicate. Whole cell extracts were prepared by TCA extraction method and samples were subjected to Western blot anlaysis using indicated antibodies. B) Wild-type (1588-4C) cells were cultured up to log phase and treated with either DMSO or curcumin (400 μM). Samples were collected after 1, 2 and 3 hr of curcumin treatment. Cultures were supplemented with iron (100 μM) after 3 hr of curcumin treatment. Again samples were collected after 1, 2, 3, 4 hr of iron supplementation. Whole cell extracts were prepared by TCA extraction method and samples were subjected to Western blot analysis using indicated antibodies. (TIF) [file pone.0059003.s002.tif]

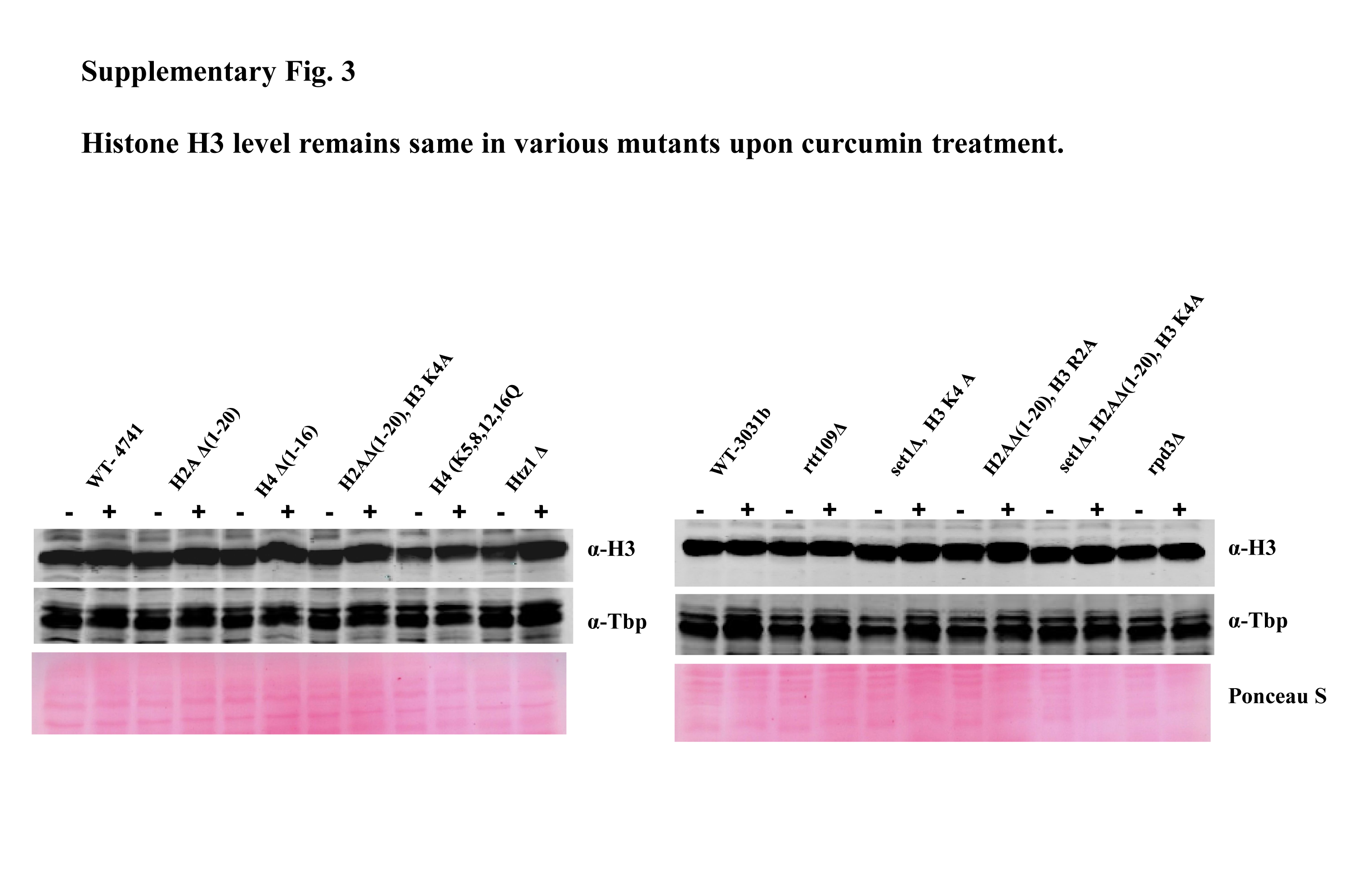

Supplement: Figure S3 — Histone H3 level remains same in various mutants upon curcumin treatment. Wild-type and various mutants shown in figure were cultured up to log-phase and treated with either DMSO or Curcumin (400 μM) for 3 hr. Whole cell extracts were prepared by TCA extraction method and samples were subjected to Western blot analysis using indicated antibodies.- and + indicates untreated and curcumin treated samples respectively. (TIF) [file pone.0059003.s003.tif]
