# Supplementary material for: Depletion of Cellular Iron by Curcumin Leads to Alteration in Histone Acetylation and Degradation of Sml1p in Saccharomyces cerevisiae
Source: PLoS One. 2013 Mar 8;8(3):e59003. doi: 10.1371/journal.pone.0059003 (PMC3592818; doi:10.1371/journal.pone.0059003)
Supplement: Table S1 — List of yeast Strains used in this study. (DOC) [file pone.0059003.s004.doc]

**Supplementary Table 1:** List of different Yeast strains used in this study

| **S.No.** | **Strain** | **Description** | **Source/Reference** |
| --- | --- | --- | --- |
| 1 | WT1588-4C | MATa ade2-1 can1-100 his3-11,15 leu2-3,112 trp1-1 ura3- | Tsukiyama lab |
| 2 | WT YCB617 | MATahis3Δ200leu2Δ::TRP1 lys2Δ202 trp1Δ63 ura3-52 | Boeke lab/ Brachmann et al. 1995 |
| 3 | *hst3*Δ | MATahis3Δ200leu2Δ1lys2Δ202 trp1Δ63 ura3-52 *hst3*Δ::TRP1 | Boeke lab/ Brachmann et al. 1995 |
| 4 | *hst4*Δ | MATahis3Δ200leu2Δ1lys2Δ202 trp1Δ63 ura3-52 *hst4*Δ::TRP1 | Boeke lab/ Brachmann et al. 1995 |
| 5 | *hst3*Δ, *hst4*Δ | MATahis3Δ200leu2Δ1lys2Δ202 trp1Δ63 ura3-52 *hst3*Δ::TRP1 *hst4*Δ::TRP1 pCEN-URA3-HST3 | Boeke lab/ Brachmann et al. 1995 |
| 6 | *hst3*Δ, *hst4*Δ, *rad24*Δ | MATahis3Δ200leu2Δ1lys2Δ202 trp1Δ63 ura3-52 *hst3*Δ::TRP1 *hst4*Δ::TRP1 *rad24*Δ::KanMX4 pCEN-URA3-HST3 | Boeke lab/ Brachmann et al. 1995 |
| 7 | UCC1111 | MATα ade2::his3Δ200 leu2Δ0 lys2Δ0 met15Δ0 trp1Δ63 ura3Δ0 adh4::URA3-TEL (VII-L)hhf2-hht2::MET15 hhf1-hht1::LEU2 pRS412 (ADE2 CEN ARS) - HHF2-HHT2 | Parthun Lab |
| 8 | *hif1* Δ | UCC1111, *hif1* Δ | Parthun Lab |
| 9 | *hat1* Δ, *hif1* Δ | UCC1111, *hat1* Δ, *hif1* Δ | Parthun Lab |
| 10 | *hat1*Δ,*hat2*Δ, Hif1 myc tag | UCC1111, *hat1*Δ,*hat2*Δ, Hif1 myc tag | Parthun Lab |
| 11 | *isw2*Δ | MATa ade2-1 can1-100 his3-11,15 leu2-3,112 trp1-1 ura3-1 *isw2*::NAT | Parthun Lab |
| 12 | BY4741 | *MAT***a** *his3Δ1 leu2*Δ*0 met15*Δ*0 ura3*Δ*0* | Samson lab |
| 13 | *taf14*Δ | BY4741 *taf14*Δ::URA3 | Samson lab |
| 14 | *rad51*Δ , *taf14*Δ | BY4741 *rad51*Δ::KanMX4 *taf14*Δ::URA3 | Samson lab |
| 15 | *rad54*Δ , *taf14*Δ | BY4741 *rad54*Δ::KanMX4 *taf14*Δ::URA3 | Samson lab |
| 16 | Mky5 Wt | W303, MAT_ ade2_1 can1_100 his3_11 leu2_3,112 trp1_1 ura3_1 LYS2 | Kobor lab/ *Mol. Cell. Biol.* 2011, 31(18):3871 |
| 17 | *htz1*Δ | MKY5, *htz1*Δ::NATMX | Kobor lab/ *Mol. Cell. Biol.* 2011, 31(18):3871 |
| 18 | H2A.Z(1-134)-3XFLAG | MKY5, H2A.Z(1-134)-3_FLAG::NATMX | Kobor lab/ *Mol. Cell. Biol.* 2011, 31(18):3871 |
| 19 | H2A.Z(1-131)-3XFLAG | MKY5, H2A.Z(1-131)-3_FLAG::NATMX | Kobor lab/ *Mol. Cell. Biol.* 2011, 31(18):3871 |
| 20 | H2A.Z(1-128)-3XFLAG | MKY5, H2A.Z(1-128)-3_FLAG::NATMX | Kobor lab/ *Mol. Cell. Biol.* 2011, 31(18):3871 |
| 21 | H2A.Z(1-124)-3XFLAG | MKY5, H2A.Z(1-124)-3_FLAG::NATMX | Kobor lab/ *Mol. Cell. Biol.* 2011, 31(18):3871 |
| 22 | H2A.Z(1-120)-3XFLAG | MKY5, H2A.Z(1-120)-3_FLAG::NATMX | Kobor lab/ *Mol. Cell. Biol.* 2011, 31(18):3871 |
| 23 | Htz1F32A | MATα lys2-128delta,leu2Δ1,his4-912delta,ura3-52,htz1::TRP1 | Yamaguchi lab/ Genes to Cells (2011) 16, 590–607 |
| 24 | Htz1 E69A | MATα lys2-128delta,leu2Δ1,his4-912delta,ura3-52,htz1::TRP1 | Yamaguchi lab/ Genes to Cells (2011) 16, 590–607 |
| 25 | Htz1 D98A | MATα lys2-128delta,leu2Δ1,his4-912delta,ura3-52,htz1::TRP1 | Yamaguchi lab/ Genes to Cells (2011) 16, 590–607 |
| 26 | Htz1 I109A | MATα lys2-128delta,leu2Δ1,his4-912delta,ura3-52,htz1::TRP1 | Yamaguchi lab/ Genes to Cells (2011) 16, 590–607 |
| 27 | Htz1 G113A | MATα lys2-128delta,leu2Δ1,his4-912delta,ura3-52,htz1::TRP1 | Yamaguchi lab/ Genes to Cells (2011) 16, 590–607 |
| 28 | H2A Δ(1-20) | MATa his3-1 leu2-0 met15-0 ura3-0 hht1-hhf1::KAN hhf-2hht2::NAT hta1-htb1::HPH hta2-htb2::NAT p[CEN LEU2 hta1Δ(1-20)-HTB1-HHT2-HHF2] | Allis lab/ PNAS2012 Apr 10;109(15):5779-84. |
| 29 | H3 Δ(1-30) | MATa his3-1 leu2-0 met15-0 ura3-0 hht1-hhf1::KAN hhf-2hht2::NAT hta1-htb1::HPH hta2-htb2::NAT p[CEN LEU2 HTA1-HTB1-hht2Δ(1-30)-HHT2-HHF2] | Allis lab/ PNAS2012 Apr 10;109(15):5779-84. |
| 30 | H4 Δ(1-16) | MATa his3-1 leu2-0 met15-0 ura3-0 hht1-hhf1::KAN hhf-2hht2::NAT hta1-htb1::HPH hta2-htb2::NAT p[CEN LEU2 HTA1-HTB1-HHT2-hhf2 Δ(1-16)] | Allis lab/ PNAS2012 Apr 10;109(15):5779-84. |
| 31 | H2A Δ(1-20),  H3 Δ(1-30) | MATa his3-1 leu2-0 met15-0 ura3-0 hht1-hhf1::KAN hhf-2hht2::NAT hta1-htb1::HPH hta2-htb2::NAT p[CEN LEU2 hta1Δ(1-20)-HTB1- hht2 Δ(1-30)-HHF2] | Allis lab/ PNAS2012 Apr 10;109(15):5779-84. |
| 32 | H2AΔ (1-20),  H3 Δ(1-5) | MATa his3-1 leu2-0 met15-0 ura3-0 hht1-hhf1::KAN hhf-2hht2::NAT hta1-htb1::HPH hta2-htb2::NAT p[CEN LEU2 hta1Δ(1-20)-HTB1-hht2Δ(1-5)-HHF2] | Allis lab/ PNAS2012 Apr 10;109(15):5779-84. |
| 33 | H3 Δ(1-5) | MATa his3-1 leu2-0 met15-0 ura3-0 hht1-hhf1::KAN hhf-2hht2::NAT hta1-htb1::HPH hta2-htb2::NAT p[CEN LEU2 HTA1-HTB1-hht2Δ(1-5)-HHF2] | Allis lab/ PNAS 2012 Apr 10;109(15):5779-84. |
| 34 | H2A Δ(1-20),  H4 S1A | MATa his3-1 leu2-0 met15-0 ura3-0 hht1-hhf1::KAN hhf-2hht2::NAT hta1-htb1::HPH hta2-htb2::NAT p[CEN LEU2 hta1Δ(1-20)-HTB1-hht2S1A-HHF2] | Allis lab/ PNAS2012 Apr 10;109(15):5779-84. |
| 35 | H2AΔ(1-20),  H3 K4A | MATa his3-1 leu2-0 met15-0 ura3-0 hht1-hhf1::KAN hhf-2hht2::NAT hta1-htb1::HPH hta2-htb2::NAT p[CEN LEU2 hta1Δ(1-20)-HTB1-hht2K4A-HHF2] | Allis lab/ PNAS2012 Apr 10;109(15):5779-84. |
| 36 | *set1*Δ,  H3 K4 A | MATa his3-1 leu2-0 met15-0 ura3-0 hht1-hhf1::KAN hhf-2hht2::NAT hta1-htb1::HPH hta2-htb2::NAT *set1*Δ::URA3 p[CEN LEU2 HTA1-HTB1-hht2K4A-HHF2] | Allis lab/ PNAS2012 Apr 10;109(15):5779-84. |
| 37 | H2AΔ(1-20),  H3 R2A | MATa his3-1 leu2-0 met15-0 ura3-0 hht1-hhf1::KAN hhf-2hht2::NAT hta1-htb1::HPH hta2-htb2::NAT p[CEN LEU2 hta1Δ(1-20)-HTB1-hht2R2A-HHF2] | Allis lab/ PNAS2012 Apr 10;109(15):5779-84. |
| 38 | *set1*Δ, H2AΔ(1-20),  H3 K4A | MATa his3-1 leu2-0 met15-0 ura3-0 hht1-hhf1::KAN hhf-2hht2::NAT hta1-htb1::HPH hta2-htb2::NAT *set1*Δ::URA3 p[CEN LEU2 hta1Δ(1-20)-HTB1-hht2K4A-HHF2] | Allis lab/ PNAS2012 Apr 10;109(15):5779-84. |
| 39 | *rpd3*Δ | MATα ura3-52 leu2-3,112 trp1-289 his3Δ1 Δ(hhf1-hht1)(hhf2-hht2) pNS329[CEN TRP1 HHF1-HHT1] *rpd3*::LEU2 | Morse lab |
| 40 | H3(K4,9,14,18,23,27Q) | MATα ura3-52 leu2-3,112 trp1-289 his3Δ1 Δ(hhf1-hht1)(hhf2-hht2) pCL460 [CEN TRP1 HHF1 hht1-3 H3 (K4,9,14,18,23,27Q)] | Morse lab |
| 41 | H4 (K5,8,12,16Q) | MATα ura3-52 leu2-3,112 trp1-289 his3Δ1 Δ(hhf1-hht1)(hhf2-hht2) pNS491 [CEN TRP1 hhf1-10 H4 (K5,8,12,16Q) HHT1] | Morse lab |
| 42 | H4 (K5,8,12,16Q) , *rpd3*Δ | MATα ura3-52 leu2-3,112 trp1-289 his3Δ1 Δ(hhf1-hht1)(hhf2-hht2) pNS491 [CEN TRP1 hhf1-10 H4 (K5,8,12,16Q) HHT1] *rpd3*:: LEU2 | Morse lab |
| 43 | H2A wild-type | MATa Δ(HTA1 HTB1) Δ(HTA2 HTB2) lys2-128 delta his3Δ200 ura3-52 pRS313[HIS HTA1 HTB1]. | Yamaguchi lab/ Genes to Cells (2011) 16, 590–607 |
| 44 | H2A-F26A | MATa Δ(HTA1 HTB1) Δ(HTA2 HTB2) lys2-128 delta his3Δ200 ura3-52 pRS313[HIS HTA1 HTB1]. | Yamaguchi lab/ Genes to Cells (2011) 16, 590–607 |
| 45 | H2A-N74A | MATa Δ(HTA1 HTB1) Δ(HTA2 HTB2) lys2-128 delta his3Δ200 ura3-52 pRS313[HIS HTA1 HTB1]. | Yamaguchi lab/ Genes to Cells (2011) 16, 590–607 |
| 46 | H2A-I103A | MATa Δ(HTA1 HTB1) Δ(HTA2 HTB2) lys2-128 delta his3Δ200 ura3-52 pRS313[HIS HTA1 HTB1]. | Yamaguchi lab/ Genes to Cells (2011) 16, 590–607 |
| 47 | H2A-G107A | MATa Δ(HTA1 HTB1) Δ(HTA2 HTB2) lys2-128 delta his3Δ200 ura3-52 pRS313[HIS HTA1 HTB1]. | Yamaguchi lab/ Genes to Cells (2011) 16, 590–607 |
| 48 | H2A K4 7G | MATa ade2-1 trp1-1 can1-100 leu2-3,112 his3-11,15 ura3 hta1-htb1:: HIS3 hta2 htb2::LEU2 carries pMP023 (CEN6 TRP1 hta1-K4,7G HTB1) H2A K4 7G | Wyric lab |
| 49 | H2A Δ(4-8) | MATa ade2-1 trp1-1 can1-100 leu2-3,112 his3-11,15 ura3 hta1-htb1:: HIS3 hta2 htb2::LEU2 carries pMP074 (CEN6 TRP1 hta1 Δ4-8 HTB1) H2A Δ(4-8) | Wyric lab |
| 50 | H2A Δ(12-20) | MATa ade2-1 trp1-1 can1-100 leu2-3,112 his3-11,15 ura3 hta1-htb1:: HIS3 hta2 htb2::LEU2 carries pMP076 (CEN6 TRP1 hta1 Δ12-20 HTB1) H2A Δ(12-20) | Wyric lab |
| 51 | H2A Δ(16-20) | MATa ade2-1 trp1-1 can1-100 leu2-3,112 his3-11,15 ura3 hta1-htb1:: HIS3 hta2 htb2::LEU2 carries pMP077 (CEN6 TRP1 hta1 Δ16-20 HTB1) H2A Δ(16-20) | Wyric lab |
| 52 | H2A S19A | MATa ade2-1 trp1-1 can1-100 leu2-3,112 his3-11,15 ura3 hta1-htb1:: HIS3 hta2 htb2::LEU2 carries pMP080 (CEN6 TRP1 hta1 S19A HTB1) H2A S19A | Wyric lab |
| 53 | WT-Rap1 | W303; rap1::LEU2; Ycp50-RAP1 | Kurtz and Shore, 1991 |
| 54 | Rap1 (∆43-279) | W303;rap1::LEU2; rap1-6 (rap1 ∆43-279 [HIS3/CEN) | Reese lab |
| 55 | Rap1 (1-716) | W303;rap1::LEU2; rap1-8 (rap1 1-716 [HIS3/CEN]) | Reese lab |
| 56 | Rap1 (1-695) | W303; rap1::LEU2; rap1-10 (rap1 1-695 [HIS3/CEN]) | Reese lab |
