# Supplementary material for: Depletion of Cellular Iron by Curcumin Leads to Alteration in Histone Acetylation and Degradation of Sml1p in Saccharomyces cerevisiae
Source: PLoS One. 2013 Mar 8;8(3):e59003. doi: 10.1371/journal.pone.0059003 (PMC3592818; doi:10.1371/journal.pone.0059003)
Supplement: Table S2 — List of oligonucleotides used in this study. (DOC) [file pone.0059003.s005.doc]

**Supplementary Table 2:** List of oligonucleotide primers used in this study

| **S.No.** | **Oligonucleotide Primer** | **Sequence (5'–3')** |
| --- | --- | --- |
| 1 | HUG1F | CCTTAACCCAAAGCAATTCTTCC |
| 2 | HUG1R | TTAGTTGGAAGTATTCTTACCAATGTC |
| 3 | RNR4F | ATGGAAGCACATAACCAATTT |
| 4 | RNR4R | GCAATTTCCTTGAATAGAGGGA |
| 5 | RNR3F | GGGTACAAAATTCTCTGAACAAA |
| 6 | RNR3R | AATGTCACAT TTCTTCTCGTCG |
| 7 | RNR2F | ATGCCTAAAGAGACCCCTTCCA |
| 8 | RNR2R | CTCGTTTTCGTTCATTCTGTTGTTC |
| 9 | RNR1F | ATTTCGTGCCCGCAGC |
| 10 | RNR1R | TTCCTCATCATCAACGATGGG |
| 11 | ACO1F | ATGTTATGGCAGGTCGTCCA |
| 12 | ACO1R | ACCCATACCAGTAGCGGAGA |
| 13 | FRE1F | GGGTCTCGTCTTCTTCTGGG |
| 14 | FRE1R | GACCCTTGCCTCGAGTTGTA |
| 15 | FET3F | TGGTCACGGACTTGACGAAG |
| 16 | FET3R | CACGGTCATTTCGTGGTCCT |
| 17 | ACT1F | CACCCTGTTCTTTTGACTGAAGC |
| 18 | ACT1R | TACCGGCAGATTCCAAACCC |
